# Supplementary material for: Evaluation of Survival, Recurrence Patterns and Adjuvant Therapy in Surgically Staged High-Grade Endometrial Cancer with Retroperitoneal Metastases
Source: Cancers (Basel). 2021 Apr 23;13(9):2052. doi: 10.3390/cancers13092052 (PMC8123054; doi:10.3390/cancers13092052)
Supplement: Supplementary file 1 [file cancers-13-02052-s001.zip › cancers-1159633-supplementary.pdf]

cancers

Supplementary Material: Survival and Recurrence Patterns of High-grade Endometrial Cancer with Retroperitoneal Metastases

Jennifer McEachron, Lila Marshall, Nancy Zhou, Van Tran, Margaux J. Kanis, Constantine Gorelick and Yi-Chun Lee

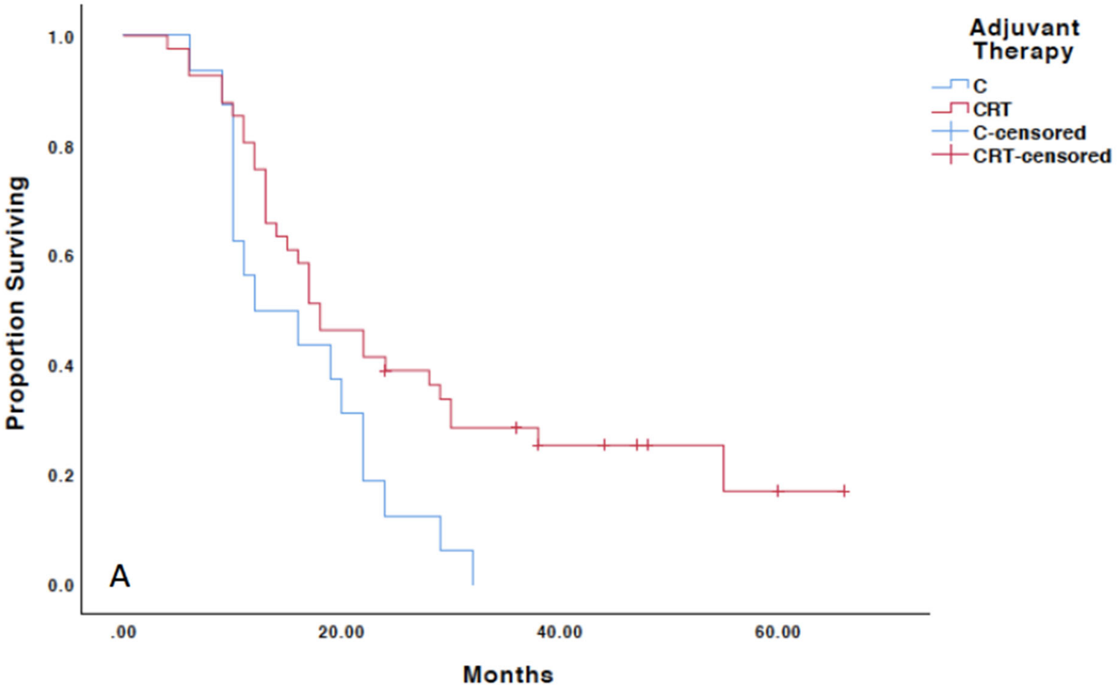

(A)

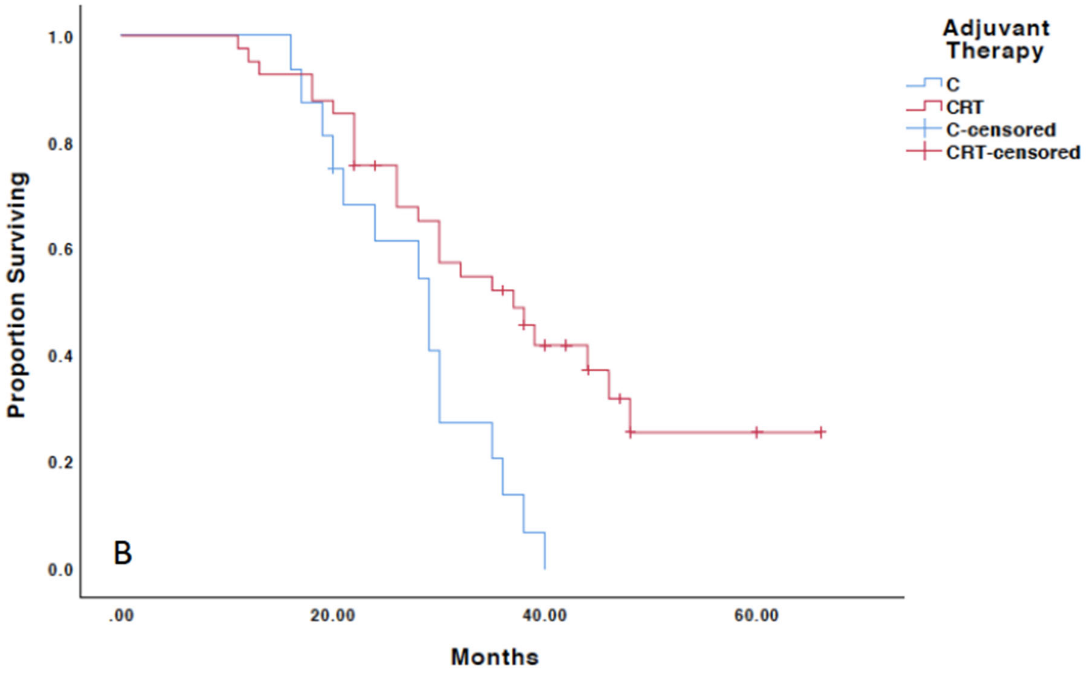

(B)

Figure S1. Kaplan – Meier survival analysis of all serous tumors by adjuvant therapy regimen; (**A)** Progression Free Survival Analysis; (**B)** Overall Survival Analysis; *CT:* Chemotherapy alone; *CRT:* Chemoradiation.

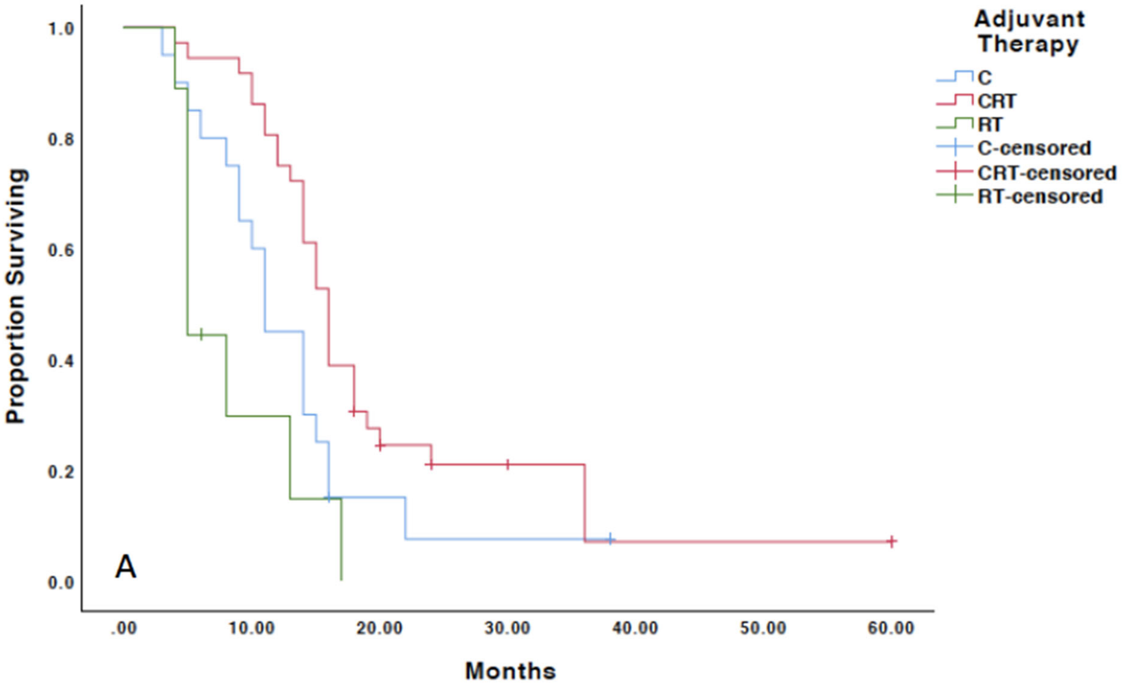

(A)

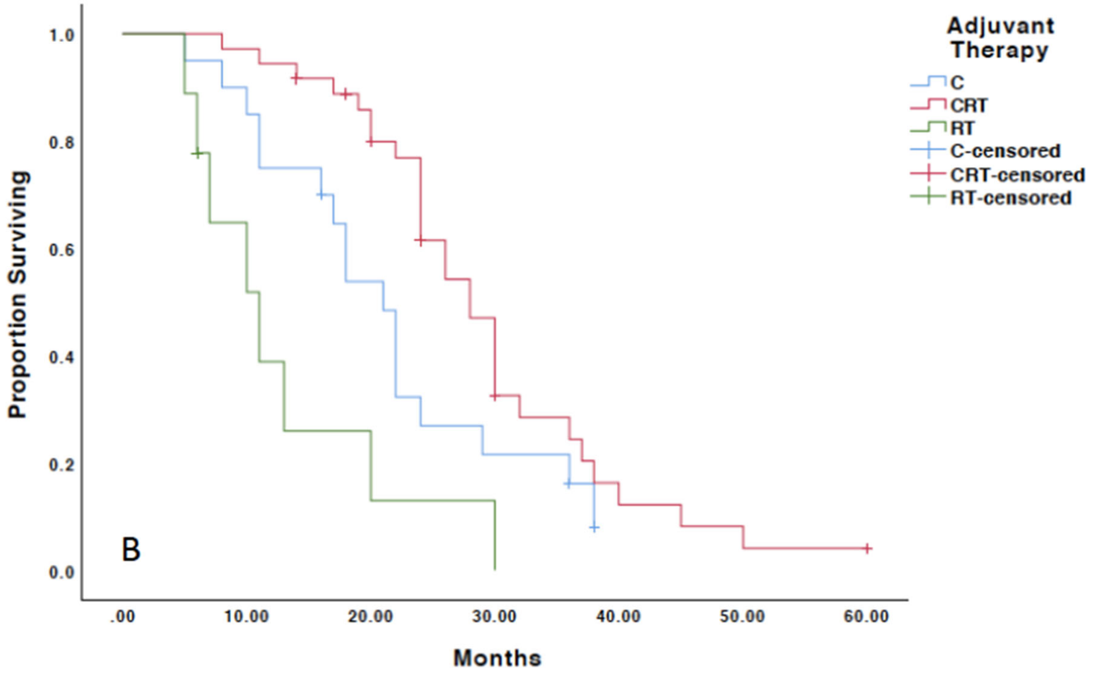

(B)

Figure S2. Kaplan – Meier survival analysis of all carcinosarcomas based on adjuvant treatment regimen; **(A)**: Progression Free Survival Analysis; **(B)**: Overall Survival Analysis; *CT:* Chemotherapy alone*; CRT:* Chemoradiation; *RT*: Radiation therapy.
